# Gonadotropic and Physiological Functions of Juvenile Hormone in Bumblebee (*Bombus terrestris*) Workers

**DOI:** 10.1371/journal.pone.0100650

**Published:** 2014-06-24

**Authors:** Hagai Shpigler, Etya Amsalem, Zachary Y. Huang, Mira Cohen, Adam J. Siegel, Abraham Hefetz, Guy Bloch

**Affiliations:** 1 Department of Ecology, Evolution, and Behavior, The Alexander Silberman Institute of Life Sciences, The Hebrew University of Jerusalem, Jerusalem, Israel; 2 Department of Zoology, George S. Wise Faculty of Life Sciences, Tel Aviv University, Tel Aviv, Israel; 3 Department of Entomology, Michigan State University, East Lansing, Michigan, United States of America; University of Sussex, United Kingdom

## Abstract

The evolution of advanced sociality in bees is associated with apparent modifications in juvenile hormone (JH) signaling. By contrast to most insects in which JH is a gonadotropin regulating female fertility, in the highly eusocial honey bee (*Apis mellifera*) JH has lost its gonadotrophic function in adult females, and instead regulates age-related division of labor among worker bees. In order to shed light on the evolution of JH signaling in bees we performed allatectomy and replacement therapies to manipulate JH levels in workers of the "primitively eusocial" bumblebee *Bombus terrestris*. Allatectomized worker bees showed remarkable reduction in ovarian development, egg laying, *Vitellogenin* and *Krüppel homolog 1* fat body transcript levels, hemolymph Vitellogenin protein abundance, wax secretion, and egg-cell construction. These effects were reverted, at least partially, by treating allatectomized bees with JH-III, the natural JH of bees. Allatectomy also affected the amount of ester component in Dufour's gland secretion, which is thought to convey a social signal relating to worker fertility. These findings provide a strong support for the hypothesis that in contrast to honey bees, JH is a gonadotropin in bumblebees and lend credence to the hypothesis that the evolution of advanced eusociality in honey bees was associated with major modifications in JH signaling.

## Introduction

Endocrine systems typically integrate multiple environmental signals and coordinate processes in multiple tissues. Hormonal regulation is genetically complex because many genes may be involved in the production, cellular responses (including threshold adjustment), and environmental regulation of endocrine signals. From an evolutionary perspective, even limited modifications in endocrine signaling pathways may affect multiple tissues and produce profound coordinated changes in morphology, physiology, or function. These characteristics make endocrine systems good candidates for accounting for extensive evolutionary novelties such as those associated with the evolution of animal societies [1]. Consistent with this premise, there is evidence implicating hormones in the regulation of processes such as caste differentiation and division of labor that are pivotal for the organization of insect societies (reviewed in [2]).

Bees provide an excellent model system for studying hormonal aspects of the evolution of sociality because phylogenetically related species show diverse forms of social living and the endocrine system of bees is better studied than that of other social insects. While most bee species are solitary, there are also many social species that exhibit diverse levels of social complexity, from small groups consisting of only a handful of individuals, to the perennial societies of honey bees and stingless bees with their complex communication systems, morphological caste system, and intricate division of labor among workers [3], [4].

The best-studied endocrine signal in the context of social organization is juvenile hormone (JH) (reviewed in [2], [5], [6]). JH regulates important functions in diverse developmental and physiological processes in insects [7]. In adult (imago) insects it typically functions as a gonadotropin that in females is best manifested in the regulation of oocyte development (oogenesis). One of the pivotal roles of JH is regulating the production of the yolk protein Vitellogenin (*Vg*) in the fat body and its accumulation in the developing oocyte [8], [9]. This JH – *Vg* – oogenesis model has been supported by studies on many, but not all, insect species studied to date [10], [11]. One of the well-studied exceptions for this model is the honey bee *Apis mellifera* in which both the highly fecund queen and egg-laying workers have low levels of JH and high levels of *Vg* [12]–[14]. Queens in which the corpora allata (CA) glands, the only known source of JH in insects [7], were surgically removed ('allatectomy') still lay eggs at a rate comparable to control queens [15]. In contrast to the positive correlation in most insects, in the honey bee JH and *Vg* levels are negatively correlated and there is evidence for a dual repressor model in which JH downregulates *Vg* expression and *Vg* suppresses JH levels [16], [17]. In worker honey bees JH and *Vg* have important non-reproductive functions, including a pivotal role in the regulation of age-related division of labor. Young worker bees that carry out in-nest tasks such as brood care have low levels of JH and high levels of *Vg* (which they use for the production of the royal jelly [18], [19]), whereas older workers typically perform foraging activities with high levels of JH and low levels of *Vg* [20]. Manipulations of JH levels by allatectomy (surgical removal of the CA glands) or treatments with JH or its analogs delayed or accelerated the time of transition from in-nest to foraging activities, respectively [21], [22]. Taken together the studies with honey bees are consistent with the premise that in this species JH has lost its gonadotrophic function during the adult stage and instead is involved in the regulation of age-related division of labor [2], [5], [23], [24].

These striking differences in JH function between solitary insects and the highly social honey, and the influence of JH on the division of labor lead to the hypothesis that modifications in JH signaling were important for the evolution of advanced sociality in honey bees [5], [24]–[26]. According to this hypothesis, JH kept its gonadotropic functions in solitary and possibly also in primitively social bees, but not in adults of the highly eusocial honey bee. Studies with solitary bees (*Osmia rufa*), facultatively social bees (*Megalopta genalis*), and the primitively eusocial bees (*Lasioglossum zephyrum* and *Bombus terrestris*) showing a positive correlation between JH levels and oocyte development lend credence this premise [27]–[31]. There is also some evidence that treatment with JH or its analogs augmented oogenesis in both the sweat bee *Lasioglossum zephyrum* [31] and the bumblebee *B. terrestris* [32], [33]. In *B. terrestris*, JH treatment accelerated oogenesis even in the presence of the queen that typically inhibits worker reproduction. Treatment with JH-I, which is not the natural JH of bumblebees [28], [29], enhanced Vg biosynthesis in the fat body of ovariectomized bumblebee gynes [34]. On the other hand, treatment with either JH-I or the JH analogue methoprene did not influence task specialization (division of labor) in the bumblebees *B. terrestris* and *B. impatiens* [35], [36]. These studies suggest that in bumblebees JH influences oogenesis but has no or little influence on division of labor. These studies however, are not sufficient to establish JH as a gonadotropin that is necessary for oogenesis and reproduction.

In this study we combined allatectomy and replacement-therapy with JH-III, the natural JH of bumblebees to rigorously test the hypothesis that JH has gonadotropic functions in the bumblebee *B. terrestris*. Our results show that JH is necessary for oocyte development and maturation and is involved in the regulation of vitellogenesis and several additional physiological processes that are associated with reproduction. We further compared our findings for *B. terrestris* to those resulting from similar JH manipulations in the honey bee, and discuss the evolution of JH signaling and sociality in bees.

## Materials and Methods

### Bees

*Bombus terrestris* colonies were purchased from Polyam Pollination Services, Yad -Mordechai, Israel. Each colony contained a queen, 5–10 workers, and brood at all stages of development. Each colony was placed in a wooden nesting box (21×21×12 cm) with a front wall and cover made of transparent acrylic plastic. The nesting boxes with the bees were housed in an environmental chamber (28±1°C; 50±5% RH) in constant darkness at the Bee Research Facility at the Edmond J. Safra campus of the Hebrew University of Jerusalem, Givat Ram, Jerusalem. The bees were fed *ad libitum* with commercial sugar syrup and fresh pollen (collected by honey bees) mixed with sugar syrup (purchased from Polyam Pollination Services). At the end of experiments we measured the marginal cell length as an index for body size. The marginal cell length is highly correlated with wing length and other body size indices, but is more reliable than wing length because it is not influenced by wing wearing and previous flight experience [37], [38]. The mean body size did not vary between bees subjected to different treatments in the three experiments described below (Table S1).

### Surgical removal of the corpora allata glands (allatectomy)

We collected newly emerged worker bees (up to 24 hours after emerging from the pupa) from several source colonies. At this age the cuticle of adult bumblebees is relatively soft and easy to manipulate. The bees had free access to sugar syrup and pollen *ad libitum* both before and after the allatectomy operation. We first anesthetized the bees on ice for 5–30 min (the variation in chilling duration was apparently due to individual differences in body size, age, and nutritional state, and because we chilled bees in groups of four) and then fixed them with molded modeling clay on an ice-chilled metal stage under a stereoscopic microscope (Nikon SMZ645, ×50). The bees were fixed with the dorsal side up and the head bent down to expose the thin neck cuticle connecting the thorax and the head. We used a fine scalpel to open a latitudinal incision in the posterior part of the head capsule, and moved the inner membrane and trachea to expose the CA glands. Using fine forceps we gently grasped each one of the corpus allatum and detached it. The entire procedure took between 2–5 minutes and the cuticle resumed its original shape and the incision appeared self-sealed within few hours after the operation. Sham-operated bees (*'Sham'*) were handled and dissected in a similar way but the CA were only touched gently and not detached. Control bees (*'Control*') were anesthetized and handled similarly, but were not operated. After treating the bees we placed them in a small cage with the other similarly manipulated workers, and let them recover overnight in an incubator (32°C, 70% RH). On the second day the surviving bees from each treatment group were assigned to groups of three, each transferred to a fresh wooden cage (12×5×8 cm). The groups were kept in the incubator for six days and then collected. Most of the mortality occurred during the first day, before assigning the bees to groups. The average survival rate for the first day in the three experiments detailed below was 50% for the allatectomized (*‘CA-‘*) bees, 80% for the sham, and 100% for the control bees. In all three experiments the survival of the CA- bees during the first day was lower compared to the Control and Sham groups (Kaplan-Meier survival analysis followed by the logrank test, Exp.1: χ^2^(2) = 18.6 p<0.001; Exp. 2: χ^2^(3) = 13 p = 0.004; Exp. 3: χ^2^(3) = 24.6 p<0.001; Fig. S1). Survival rate in the cages during day two to seven was better for the CA- bees (70%), and was similar to that of the Sham, and Control bees in experiments 2 and 3 (80%, 95%, respectively; Logrank test; Exp. 1: χ^2^(2) = 6.3 p = 0.04; Exp. 2: χ^2^(3) = 0.78 p = 0.85; Exp. 3: χ^2^(3) = 6.1 p = 0.1).

### Replacement therapy

For replacement therapy (*'CA-+JH'*) we chilled 2 days old allatectomized bees (24 hours after the surgery) on ice for 3–5 minutes, and when anesthetized treated topically with 70 ng JH-III (Sigma, cat #: J-2000) dissolved in 3.5 µl Dimethylformamide (DMF, J.T Backer, cat #: 7032), giving a final concentration of 20 ng/µl. The JH solution was applied to the dorsal part of the thorax [33]. This treatment protocol is based on previous studies with honey bees and our studies with bumblebees [33],[39]. Sham and CA- bees where similarly handled and chilled, but only treated with the vehicle (3.5 µl DMF). The control group was chilled, but otherwise untreated. Following treatment the bees were placed back into their cage. Survival was similar for the CA-, and the CA-+JH bees (Fig. S1).

### Measurement of hemolymph JH titers

Hemolymph samples were collected from bees of the CA-, Sham, and Control treatments at seven day of age. The bees were chilled on ice for 3–5 min and fixed with modeling clay on a wax base surgical plate with the dorsal parts facing up. We opened a small incision in the membrane connecting the head and the thorax and drew a hemolymph sample using a 10 µl glass capillary tube (Drummond, Cat #: 5-000-1001). We collected 1–7 µl of hemolymph from each bee, immediately transferred the sample into a 5 ml glass vial containing 500 µl of HPLC grade acetonitrile (Bio-Lab, Cat # 01203501), and secured the vial with a Teflon-lined cap. The samples were kept frozen (−20°C) until shipped on dry ice to Michigan State University for analysis. We measured JH titers using a radioimmunoassay as described in [29]. The samples were coded such that the individual performing the RIA was blind to the treatment. We used one-way ANOVA followed by LSD post-hoc test to compare JH titers for bees subjected to different treatments. We used SPSS 17.0 software (IBM) for all statistical analyses.

### Chemical analyses of Dufour's glands

Seven-day-old bees from the CA-, Sham and Control groups were collected and their ovaries were removed (see below). The rest of the carcass was kept frozen (−20°C) until shipped on dry ice to Tel-Aviv University for analysis. Dufour's gland was separated from the sting apparatus and extracted in 50 µl pentane containing 1 µg eicosane as internal standard. Chemical analyses were performed by gas chromatography using DB-1 fused silica capillary column (30 m×0.25 mm ID) under a temperature program from 170°C to 300°C at 4°C/min. Compounds identity was ascertained by gas chromatography/mass spectrometry (GC/MS) by comparing retention times and mass fragmentation of synthetic standards [40]. Compound quantification was achieved by GC peak integration compared to the internal standard under the same chromatographic conditions. We used one-way ANOVA followed by LSD post-hoc test to compare the glands' total secretion and ester contents.

### Assessing ovarian state

Bees were chilled on ice and fixed on a wax dissecting plate under a stereo microscope (Nikon SMZ645). We opened two lateral incisions in the abdomen, immersed the internal organs in bee saline [41], and dissected out the ovaries. The ovaries were placed in a drop of saline on a microscope slide and the length of 4–8 terminal (basal) oocytes was measured with an ocular ruler under a dissecting microscope (X10). The mean length of the terminal oocytes was used as an index of ovarian state for each bee [29]. We compared the average ovarian development between the groups using Kruskal–Wallis test followed by Conover post-hoc tests.

### Measurement of *Kr-h1* and *Vg* fat body mRNA levels

We used qPCR to examine the influence of JH on fat body *Vg* and *Kr-h1* mRNA levels. We collected 7-day-old bees from the four experimental groups described in experiment 3 (see below; CA-, Sham, Control and CA-+JH^2^) and chilled them on ice. The abdomen was opened and we removed the ovaries (and measured terminal oocyte size), gut, and other internal organs, leaving the fat body and the dorsal cuticle. We flash-froze the fat body in liquid nitrogen and saved it in an ultra-low freezer (−80°C). The total RNA from the dissected tissue was extracted using the Invisorb Spin Tissue RNA Mini Kit (Invitek GmbH, Berlin), and quantified via a ND-1000 Spectrophotometer (NanoDrop Technologies). cDNA was synthesized from 250 ng of RNA. To estimate the level of gene expression we used an ABI7000 sequence detector and a SYBR green detection protocol (Applied Biosystems [33]). *Elongation Factor 1 a* (*Ef1a*) was used as a control housekeeping gene based on previous studies with honey bees and bumblebees showing that its transcript levels do not vary with age or task [33], [39], [42]. Quantification was based on the number of PCR cycles required to cross a threshold of fluorescence intensity (Ct). For statistical analyses we used the ΔCt values which are normally distributed. One-way ANOVA followed by the LSD post-hoc test were used to compare transcript levels between treatment groups. For graphical representation we normalized the ΔCt values relative to the lower sample. The fold differences in relative expression levels were depicted as mean ± SE (using the 2^−ΔΔCt^ method according to the ABI User Bulletin 2). The sequences of the primers used to PCR amplify the *B. terrestris Vg*, *Kr-h1* and *Ef1a* cDNA are presented in Table S2.

### Immunoblots for hemolymph VG protein

We used immunoblots ("Western blots") to assess the influence of JH on VG hemolymph protein levels. We collected a 2.5–7 µl hemolymph sample from each bee from the 4 experimental groups studied in Experiment 3 (CA-, Sham, Control and CA-+JH^2^) as described above. We immediately dissolve the hemolymph in Laemmli Sample Buffer (LSB) plus 1% of protease inhibitor cocktail 17 (PIC, Sigma, Cat#: P8340) and 0.2% EDTA (J.T Baker cat#: V49658) in a ratio of 1/5 and incubated at 95°C for 5 minutes. We analyzed the samples using SDS 7% PAGE. We used rabbit anti- *Apis mellifera* VG antibodies [19], at 1∶3000 and goat anti-rabbit secondary antibodies (Thermo Scientific, Cat#: 31460) at 1∶50000. Detection of the protein was performed with EZ-ECL – chemiluminescence detection kit for horseradish peroxidase (Biological Industries cat.#: 20–500). For gel photographing and analyses we used a LAS-1000 Luminescent Image Analyzer (FujiFilm). To predict the size of the BtVG protein we first used ExPASy (http://web.expasy.org/translate) to translate nucleotide mRNA sequence to a protein sequence, and multiple sequence alignment (ClastalO) with cloned Vg mRNA of related bumblebees that are available in the NCBI database (*Bombus ignitus* and *Bombus hypocrita*). For estimating the predicted protein size in Daltons we used the ProtParam (http://web.expasy.org/protparam) and PeptideMass (http://web.expasy.org/peptide_mass) protein size calculators. We estimate the molecular size of stained proteins by comparing their gel migration distance to that of proteins of a known size ladder (Fementas, cat#: SM 1851).

### Experiment 1: Effect of allatectomy on hemolymph JH titers, ovarian development and Dufour's glands secretion

Callow workers were collected from 6 colonies and divided randomly to three experimental group, *'CA-'*, *'sham'* and *'control'*. Each group contained three workers that received the same treatment and were placed in small wooden cages (12×5×8 cm) in an incubator (32°C, 70% RH). The bees received sugar syrup and pollen cake *ad libitum*. We inspected the cages at least once a day. Only groups in which at least two bees survived to the end of the experiment were used in our analyses. On day seven, we collected the bees, and measured for each hemolymph JH titers, ovarian development, and Dufour's gland secretion composition. We chose to sample the bees at the age of seven days because at this age queenless workers show the entire range of oocyte development with little or no signs of egg absorption [28], [43]. The experiment was conducted during May – July 2010.

### Experiment 2: Influence of JH on ovarian development, egg-laying, and wax secretion

Callow workers were collected from 8 colonies (about 20–30 bees per colony, collected over several days) and divided to experimental groups as in Experiment 1. Half of the CA- bees were treated once with JH-III as described above (CA-+JH^1^). On day seven we collected the bees, removed their ovaries, and assessed their ovarian state. At the end of the experiment we opened all egg-cups and counted the number of eggs in each cage. We used two indices for estimating the amount of wax secreted during the experiment. First, we counted the number of wax pots and cups in each cage. Second, we scraped the wax from the bottom and the sides of each cage using a fine scalpel and forceps and weighed it using fine scale with accuracy of 1 mg (Mettler Toledo; AB54–5). We used Kruskal–Wallis tests followed by Conover post-hoc tests to compare the number of eggs, wax cells, and wax weight. This experiment was conducted during April – May 2011.

### Experiment 3: Influence of JH on ovarian development, egg-laying, *Vg* and *Kr-h1* fat body mRNA expression, hemolymph VG protein levels, wax secretion, and wax cells number

The experimental design was similar to that described for Experiment 2. In order to increase the effectiveness of the replacement therapy we used two consecutive JH treatments delivered when the bees were at day 2 and 4 after emergence (CA-+JH^2^). We determined the influence of JH on ovary state, egg laying, and wax secretion as described above. In addition, we measured fat body *Vg* and *Kr-h1* mRNA levels using the qPCR detailed above, and immunoblot to estimate hemolymph VG protein levels for bees from the 4 treatment groups. All the measurements in this experiment were taken from the same focal bees. The experiment was conducted during April-May 2012, and the molecular analyses during the following months.

## Results

### Effect of allatectomy on hemolymph JH titers (Exp. 1)

**Figure 1:**
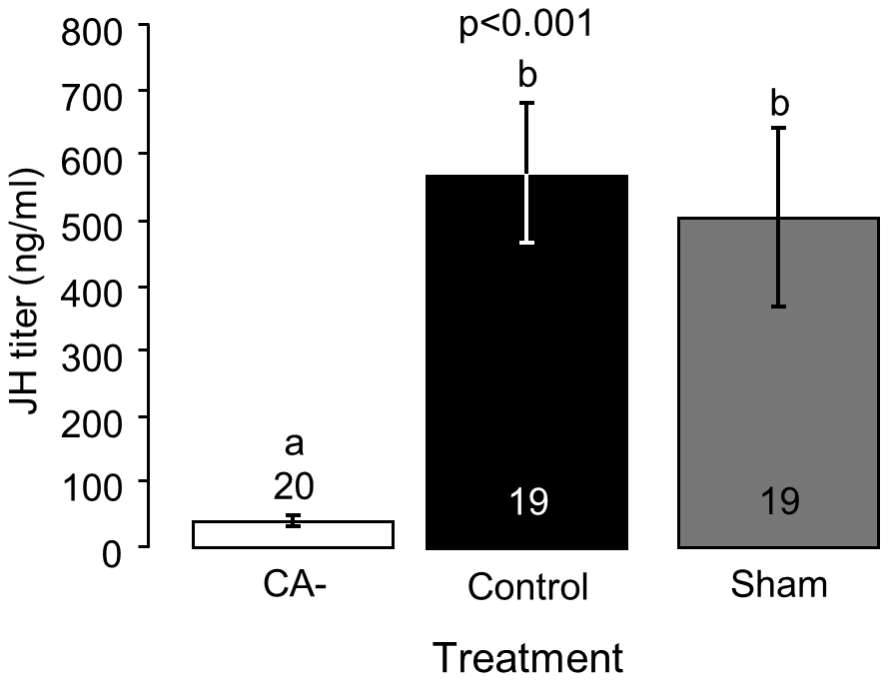
The influence of allatectomy on hemolymph JH titers. Bumblebee workers receiving the same treatment were housed in groups of three in small cages without a queen. JH titers were determined at the age of 7 days with a JH-III specific radioimmunoassay. Shown are mean ± SE, and sample size within or above the bars. The p-value summarizes the results of one-way ANOVA; groups with different letters differ significantly in a LSD post-hoc test (p<0.05). CA-  =  allatectomized bees; Sham  =  sham operated bees, Control  =  control bees.

Allatecomized bees (CA-) had an order of magnitude lower levels of hemolymph JH titers (39±8 ng/ml, n = 20) compared to control (453±79 ng/ml, n = 19) and sham operated (327±65 ng/ml, n = 19) bees (one-way ANOVA, F = 8.83, p<0.001; Fig 1). JH titers were similar for the sham treated and control bees (LSD post-hoc p>0.05). These results are consistent with the hypothesis that the CA glands are the only source of JH in the bumblebee *B. terrestris*.

### Influence of JH on oogenesis and egg-laying (Exps. 2 and 3)

**Figure 2:**
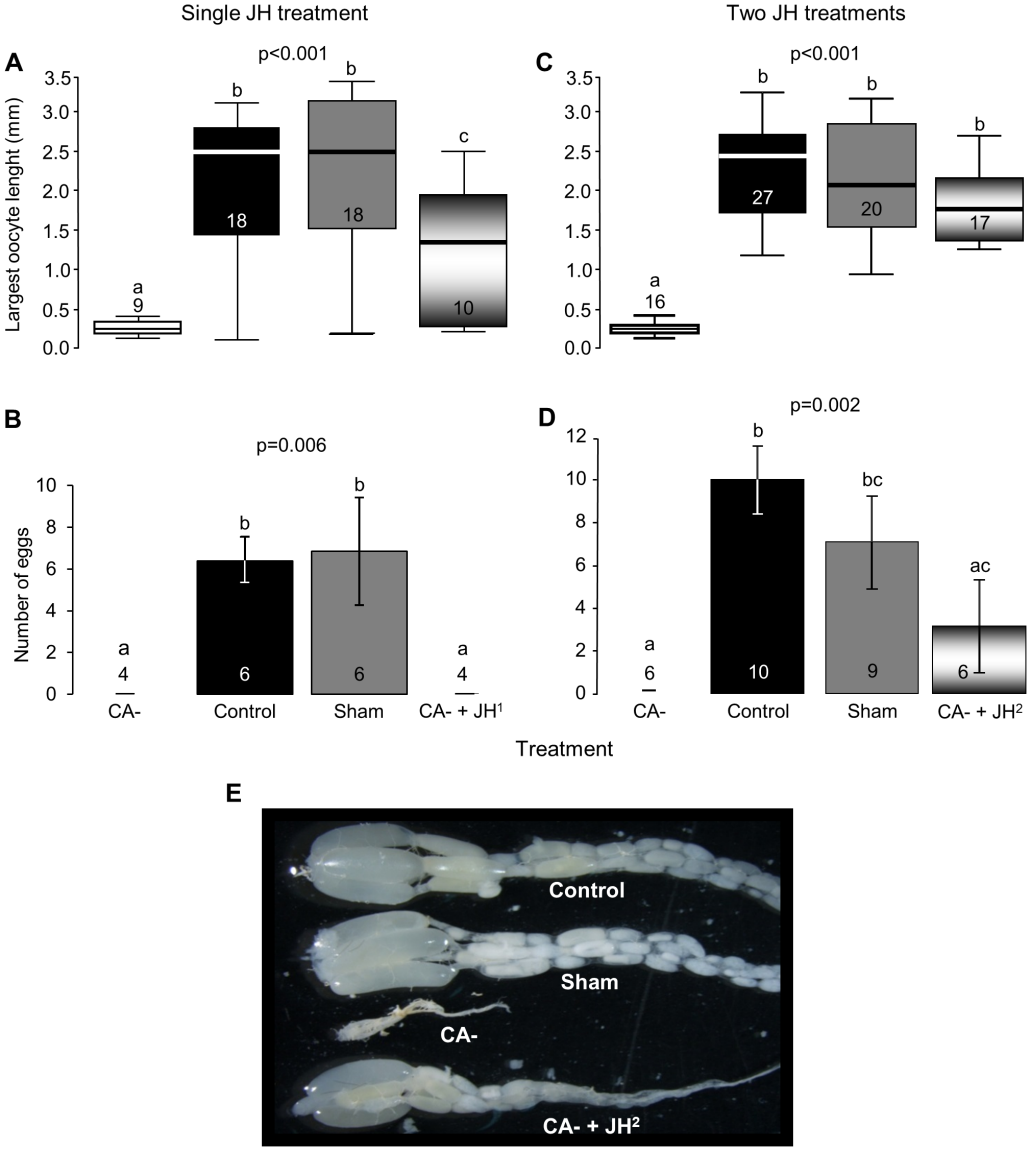
The influence of JH on worker fertility. **A.** The influence of JH on oocyte development. The values are median and 95% confident intervals for the length of the terminal oocyte. The replacement therapy included a single topical treatment with JH-III (CA-+JH^1^). **C**. Same as A. but the replacement therapy included two successive treatments with JH-III (CA-+JH^2^). **B**. The influence of JH on egg-laying. The values are mean ± SE number of eggs laid in a cage accommodating a queenless group. **D**. Same as B. but the replacement therapy included two successive treatments with JH-III. Sample size is shown within or above bars and depicts the number of bees in A and B, and the number of cages (groups) in C and D. The p-value summarizes the results of Kruskal-Wallis test; groups with different letters differ significantly in a Conover post-hoc test (p<0.05). **E**. A photograph of representative ovaries from Experiment 3 (Summarized in panel C). For additional details see legend to Fig. 1.

In Exp. 2, ovaries of allatectomized bees contained small oocytes at basal stages of development (mean terminal oocyte length 0.32±0.15 mm, n = 9) compared to bees from the other three experimental groups which also had large oocytes (Kruskal-Wallis test, χ^2^(3) = 12.5, p = 0.005; Fig. 2A). Replacement therapy with a single JH treatment (CA- + JH^1^) resulted in a significant increase in mean terminal oocyte length (1.33±0.29 mm, n = 10) relative to the CA- group, but oocyte length was nevertheless shorter than in sham-treated (2.15±0.24 mm, n = 18) or control (2.17±0.24 mm, n = 18) bees (Conover post-hoc test p<0.05). Ovarian state was similar for the sham-treated and the control bees. Eggs were laid by the control (6.4±1.2 eggs per cage, n = 6 cages) and sham-treated bees (6.9±2.5, n = 6), but not by the CA- (n = 4) or the CA- + JH^1^ (n = 4) bees (Kruskal-Wallis test, χ^2^(3) = 12.4, p = 0.006; Fig 2B).

In Exp. 3, allatectomized workers subjected to replacement therapy with two JH treatments (CA- + JH^2^) had ovaries as developed (2.24±0.17 mm, n = 17; Figs 2C and E) as in the control (2.71±0.12 mm, n = 27) and sham operated (2.55±0.2 mm, n = 20) bees (p>0.05), and with significantly larger oocytes compared with the CA- bees (0.45±0.19 mm, n = 16; Kruskal-Wallis test χ^2^(3) = 37.3, p<0.001 followed by Conover post-hoc test p<0.05; Fig 2C). As in the previous experiments (Exp. 2, see Fig. 2; Exp. 1, see Fig. S2), allatectomized bees had undeveloped ovaries containing only oocytes at basal stages. Eggs were laid in cages of the control (10±1.6 eggs per cage, n = 10), sham (7.1±2.1, n = 9), and the CA-+ JH^2^ groups (3.1±2.2, n = 6; egg were laid in only half of the cages), but not in cages with CA- (n = 6) bees (Kruskal-Wallis test, χ^2^(3) = 14.5, p = 0.002; Fig 2D). The average number of eggs laid in the CA-+JH^2^ group was lower than in the control (Conover post-hoc test, p<0.05), but not the sham operated group (Conover post-hoc test, p>0.05). The difference in numbers of eggs laid between the CA-+JH^2^ and the CA- bees, and between the sham and control groups were not statistically significant (Conover post-hoc test, p<0.05).

### The influence of JH on *Vg* expression in the fat body and hemolymph (Exp. 3)

**Figure 3:**
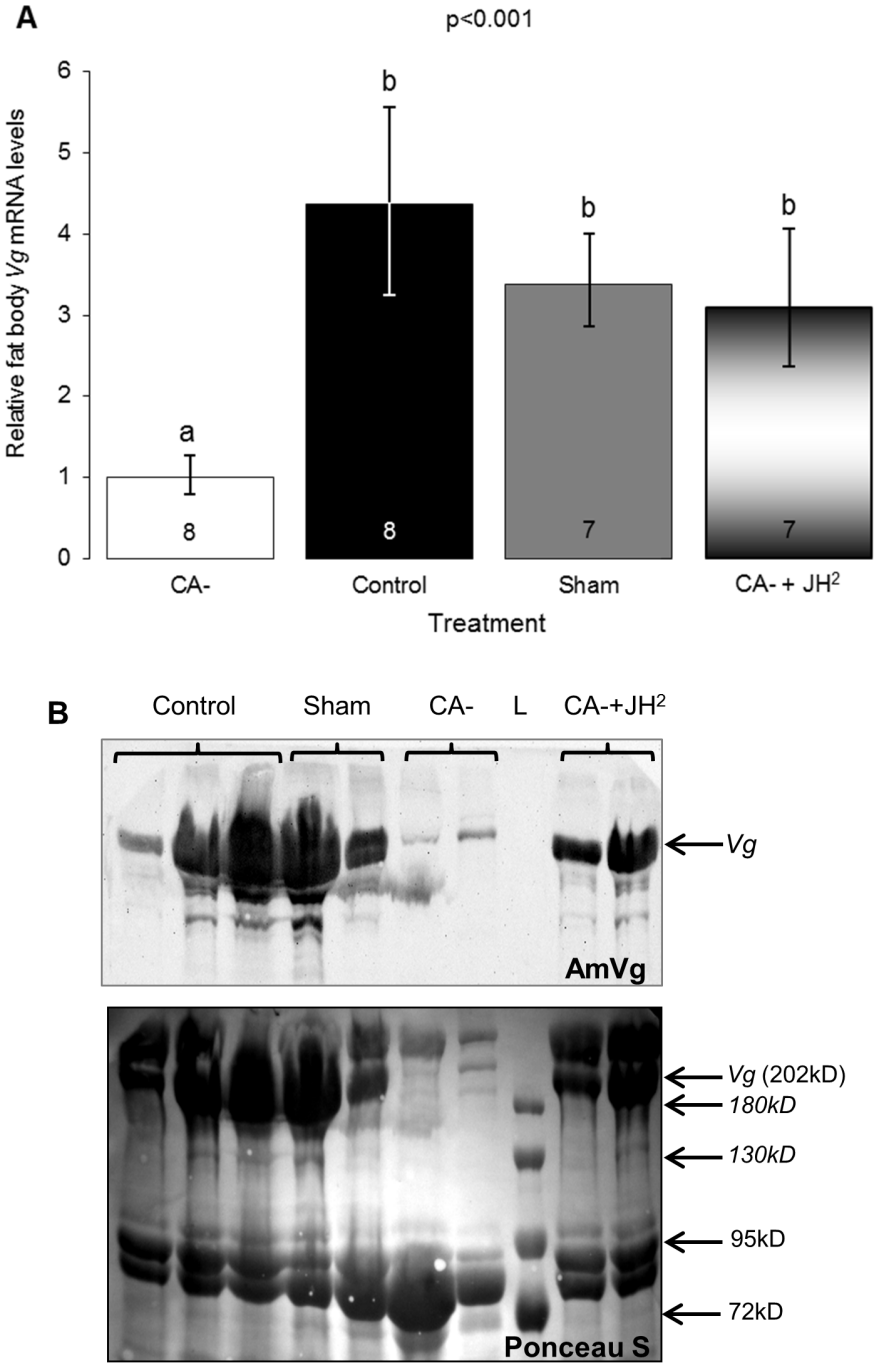
The influence of JH on vitellogenin levels. **A.** Vitellogenin (*Vg*) transcript abundance in the fat body (mean ± SE; sample size within bars). The p-value summarizes the results of one-way ANOVA; groups with different letters differ significantly in a LSD post-hoc test (p<0.05). **B**. VG protein levels in the hemolymph. The upper blot shows immunostaining with an antibody directed against the *Apis mellifera* VG protein. The lower panel shows non-specific protein staining of the same blot with Ponceau S. Each column contains data from a single bee. For additional details see legends to Figs. 1 and 2.

Fat body *Vg* mRNA levels were three to four times lower in the allatectomized bees (CA-, n = 8) compared with the control (n = 8) and the sham operated bees (n = 7; one-way ANOVA F = 9.7, p<0.001, LSD post-hoc test p<0.01). Replacement therapy with two JH treatments (CA- + JH^2^) recovered fat body *Vg* mRNA to levels similar to that in bees from the control and the sham operated groups (LSD post-hoc test p>0.05, Fig 3A). Qualitative assessments of JH influence on hemolymph VG protein levels are consistent with the findings for transcript abundance. In the control and sham operated bees there were strong bands corresponding to a protein of about 202 kD, the predicted size of the *Bt*VG protein. This band was stained strongly in the immunoblot and was also clearly identified in gels stained with the non-specific protein dye Ponceau S. By contrast, the staining in the CA- bees was very weak. The staining for bees receiving replacement therapy (CA-+JH^2^) was stronger than in the CA- bees and almost as strong as in bees from the control and sham operated groups (Fig 3B). These results are consistent with the hypothesis that JH up regulates Vg expression.

### The influence of JH on *Kr-h1* mRNA expression in the fat body (Exp. 3)

**Figure 4:**
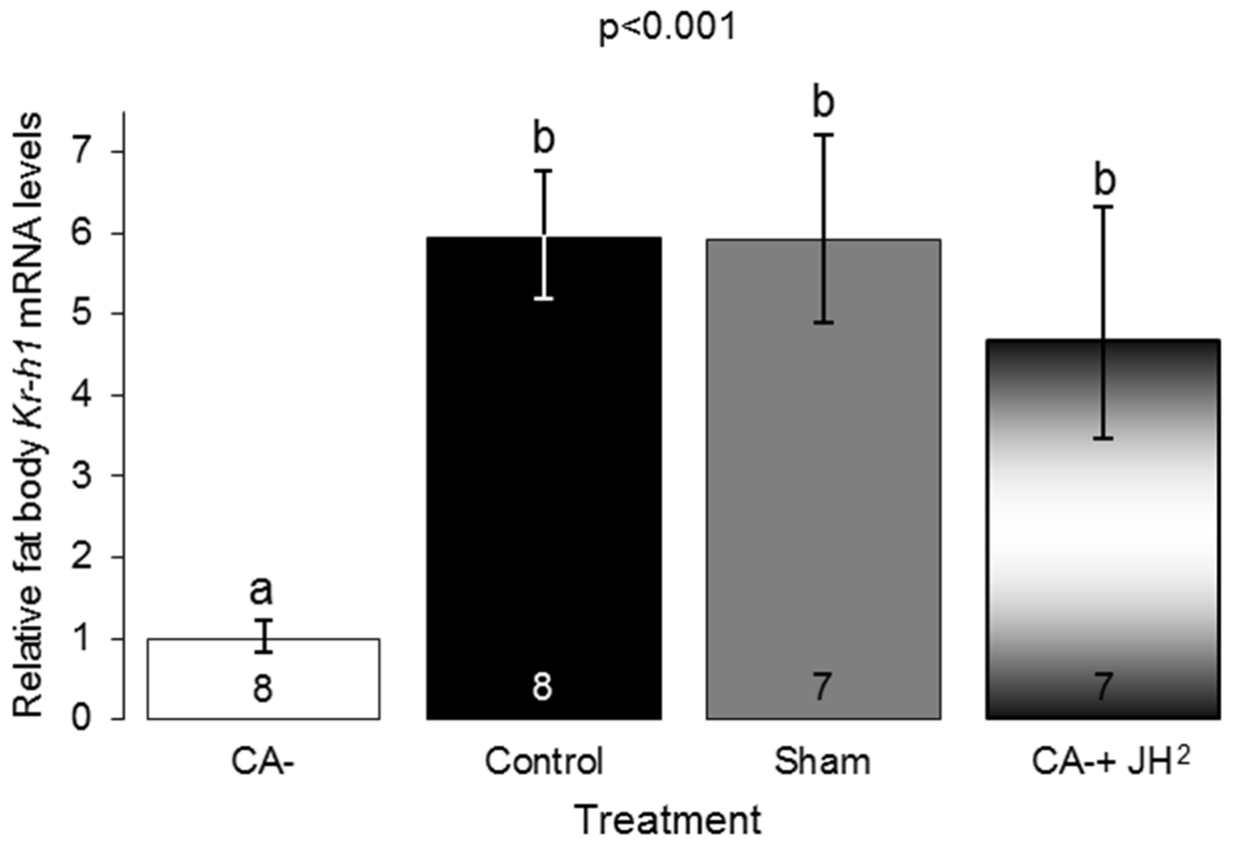
The influence of JH on fat body *Kr-h1* mRNA levels. For plot details see legend to Fig. 3A.

Fat body *Kr-h1* mRNA levels were approximately six times lower in allatectomized bees (CA-, n = 8) compared with control (n = 8) and sham operated bees (n = 7; Fig. 4; one-way ANOVA, F = 20.3, p<0.001; LSD post-hoc test, p<0.01). Replacement therapy with two JH treatments (CA-+JH^2^, n = 7) resulted in a full recovery of mRNA levels similar to that in the control and the sham operated bees (LSD post-hoc test, p>0.05, Fig. 4). This finding supports the hypothesis that in *B. terrestris Kr-h1* expression is regulated by JH.

### The influence of JH on wax secretion and wax cup construction (Exps. 2 and 3)

**Figure 5:**
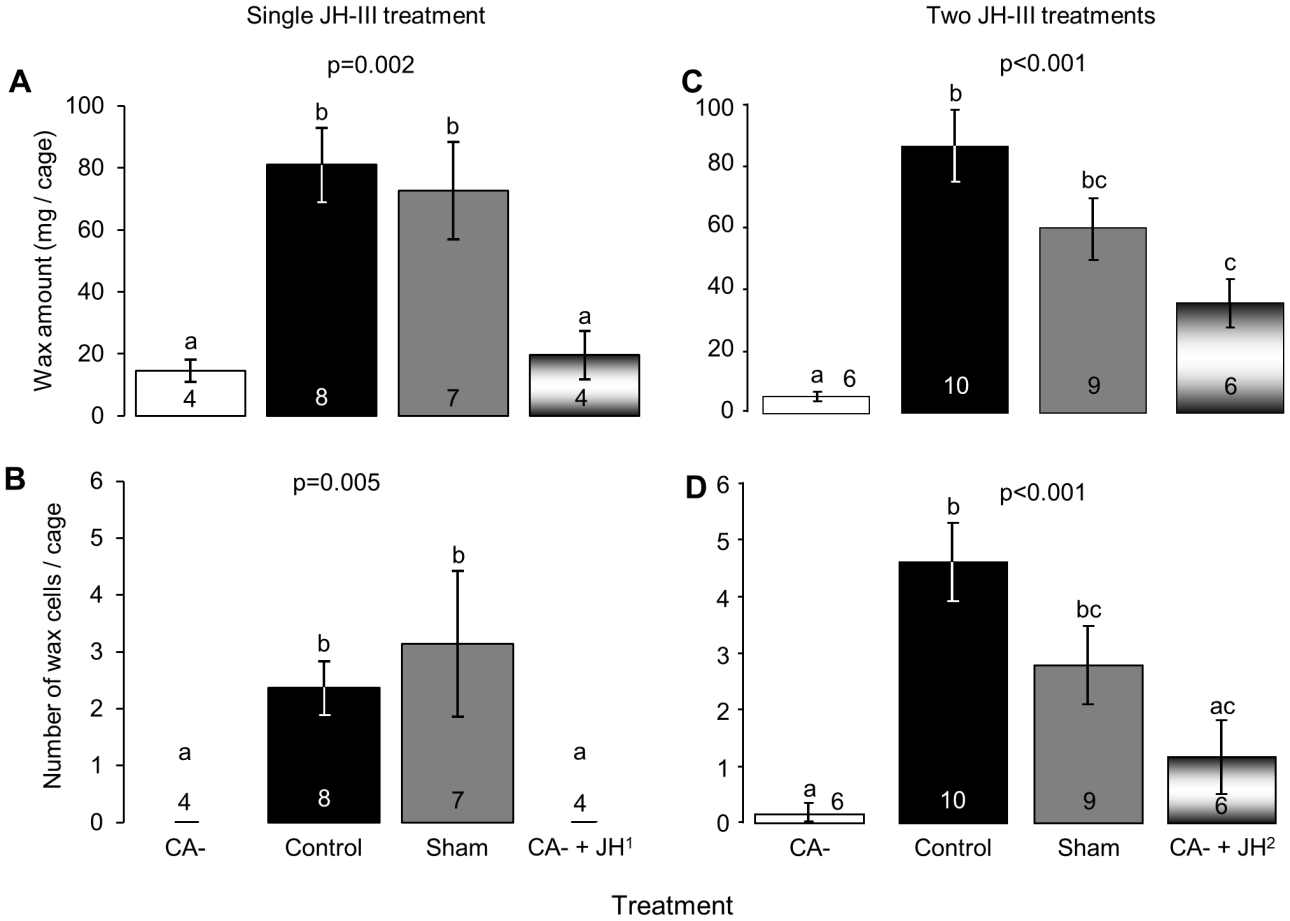
The influence of JH on wax secretion. **A and C.** The amount of wax secreted; **B and D**. the number of wax pots and cells constructed in a cage during the experiment. The replacement therapy included a single JH-III treatment in A and B, and two successive treatments in C and D. The p-values summarize the results of Kruskal-Wallis test; groups with different letters differ significantly in a Conover post-hoc test (p<0.05) test). Other details as in Fig. 2.

In Exp. 2 allatectomized bees secreted significantly less wax (CA-, 14±3 mg, n = 4 cages) compared to control (80±10 mg, n = 8) and sham-treated (72±15 mg, n = 7) bees. A single replacement therapy (CA-+JH^1^) did not recover wax secretion to the levels measured for untreated bees (19±7 mg, n = 4; Kruskal-Wallis test, χ^2^(3) = 14.2, p = 0.003; Fig. 5A). Bees from the control and sham operated, but not the CA- or single replacement therapy treatment groups built wax cells (Kruskal-Wallis test χ^2^(3) = 12.5, p = 0.005; Fig 5B). Replacement therapy with two JH treatments (CA-+JH^2^) resulted in partial recovery measured as a significant increase in both wax secretion (36±8 mg, n = 6 cages, Fig. 5C) and cell construction (1.2±0.7, n = 6, Fig. 5D) relative to the CA- bees (wax: 7.4±1 mg; cells: 0.2±0.1, n = 6). However, wax secretion and cell construction in the CA-+JH^2^ groups were still lower than in cages of the control (wax: 86±11 mg; cells: 4.6±0.7, n = 10) and sham operated (wax: 59±10 mg; cells: 2.8±0.7, n = 9) bees (Kruskal-Wallis test, χ^2^(3) = 14.07, p<0.001, Figs 5C and 5D). We found similar effect of allatectomy on wax secretion also in experiment 1 (Fig. S3). These findings suggest that JH affects wax secretion in the bumblebee *B. terrestris*.

### The effect of allatectomy on Dufour's gland secretion (Exp. 1)

**Figure 6:**
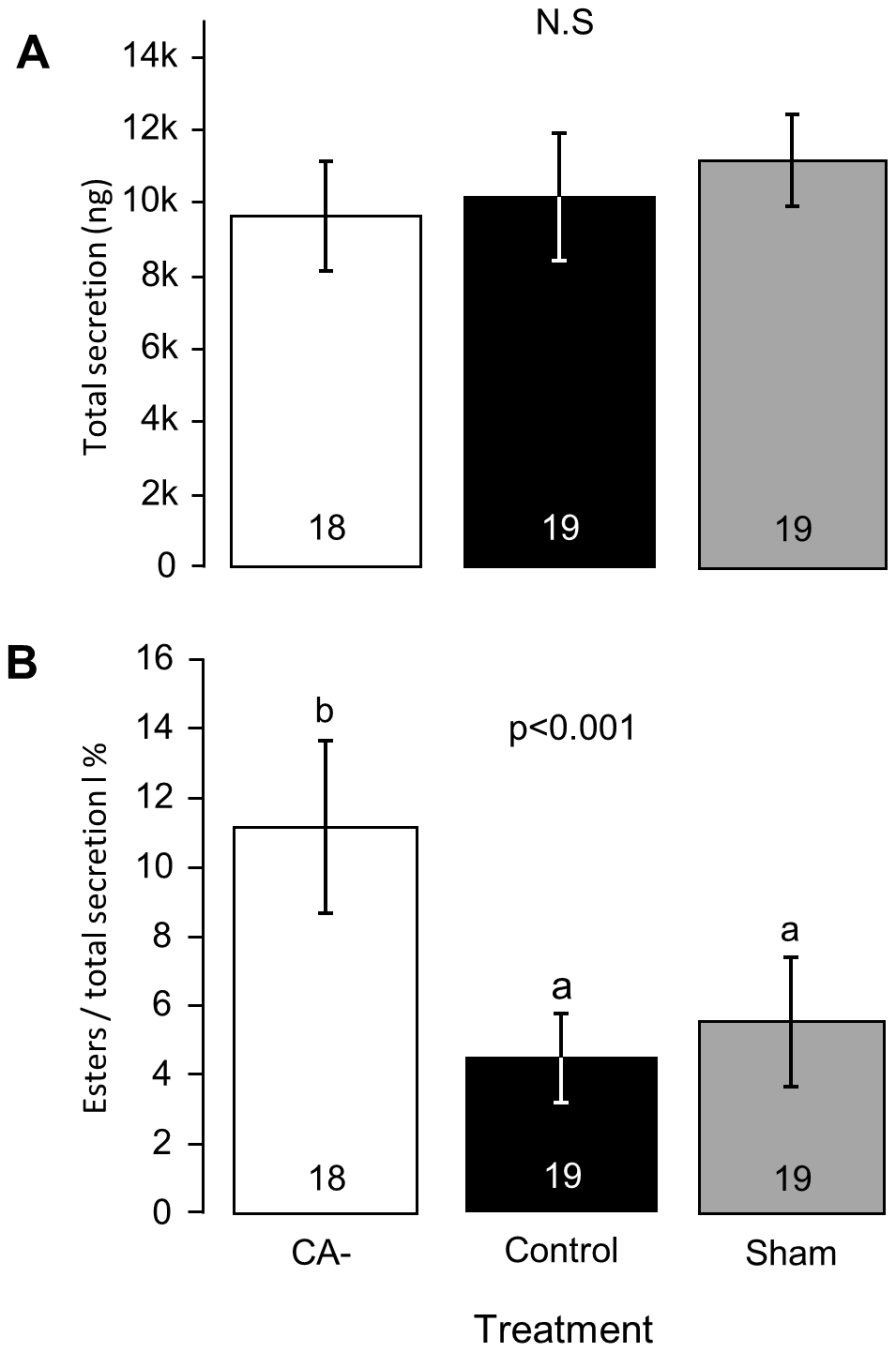
The influence of allatectomy on ester amounts in the Dufour's gland secretion. **A.** Total amount of secretion. **B**. The relative amounts of esters out of the Dufour's gland secretion. The Dufour's gland secretion was analyzed at the age of 7 days using gas chromatography/mass spectrometry (GC/MS). Shown are the mean ± SE, sample size within bars. The p-value summarizes the results of one-way ANOVA; groups with different letters differ significantly in a LSD post-hoc test (p<0.05).

The total secretion was similar in allatectomized, control, and sham operated bees (one-way ANOVA F = 0.51, p = 0.6, Fig. 6A). However, the ester fraction was higher in bees without JH (CA-, n = 18) compared with the control (n = 19) and the sham groups (n = 19; one-way ANOVA F = 9.95, p<0.001, LSD post-hoc test, p<0.01; Fig. 6B). The levels were similar for the control and the sham operated bees (LSD post-hoc test, p = 0.58). These results suggest that JH is involved in regulating the exocrine activity of the Dufour's gland.

## Discussion

Our results show that JH is necessary for oocyte development and egg-laying in *B. terrestris* workers and therefore provide the strongest available support for the hypothesis that JH functions as a gonadotropin in the bumblebee *B. terrestris*. These findings for the bumblebee contrast with evidence that in honey bees JH does not have a similar function and highlight an evolutionary enigma relating to the role of JH signaling pathways in the evolution of sociality in bees.

Our findings support and extend previous studies showing positive correlations between JH and oocyte development in *B. terrestris*, as well as acceleration of oocyte growth in bees treated with JHI, JHIII, or JH analogues [28], [29], [32], [33], [44], [45]. The current study is the first to include manipulations that reduce JH levels and thus demonstrates that JH is necessary for bumblebee reproduction. Hemolymph JH titers were strictly reduced in allatectomized bees (Fig. 1), showing that the CA removal protocol was effective, and suggesting that that the CA glands are the only source of JH in bumblebees, as was also shown for other insects [46]. In all conducted experiments, allatectomized bees had undeveloped ovaries containing only oocytes at basal developmental stages (Figs. 2A, 2C, Fig. S1). This finding is notable given that the bees were kept in small queenless groups, a social environment in which ovarian development is typically rapid [28], [43], [47]–[49]. The stronger influence of two compared to a single replacement JH treatments (Fig. 2) shows that the inhibition of oocyte development in allatectomized bees was due to the lack of JH and not other factors that may have been compromised by the allatectomy surgery. Allatectomized bees also showed reduced *Vg* transcript levels in the fat body, and protein levels in the hemolymph; the recovery of these reductions by replacement therapy (Fig. 3) are consistent with the hypothesis that JH regulates oogenesis by activating the production of the yolk protein *Vg* in the fat body [10]. The strong and opposing influences of allatectomy and replacement therapies on the fat body expression of *Kr-h1* suggest that this transcription factor mediates at least part of the influences of JH on the fat body. This premise is also consistent with studies showing that JH stimulates brain *Kr-h1* expression in *B. terrestris* [33](Shpigler and Bloch unpublished data) and that it is a canonical component of JH signaling pathways in insects [50]–[53]. Based on these findings we propose that in *B. terrestris* JH regulates oogenesis by activating *Vg* transcription in the fat body in a signaling pathway involving the JH-responsive transcription factor *Kr-h1*. Following this transcriptional activation the VG protein is released into the hemolymph and transported to the ovaries in which it is deposited in the developing oocytes. It is yet to be determined whether JH is also involved in additional processes (e.g. *Vg* intake into the developing follicles) that are necessary for oocyte development [10], [11].

Our study further shows that the influence of JH on bumblebee reproduction is not limited to ovary activation. Wax secretion was severly compromized in allatectomized bees, and this was partialy recuperated by replacement therapy with two JH treatments (Figs. 5C, 5D). Bumblebees use the wax they secrete for building pots and cells, including egg and brood cells and therefore, wax secretion needs to be coordinated with other reproductive activities. Indeed, allatectomized bees did not build pots and wax cells (Fig. 5 and Fig. S3). It is yet to be determined whether this reflects a direct influence of JH on wax glands or an indirect influence mediated by factors secreted by the developing oocyte. The failure of two replacement JH treatments to recover wax secretion to the levels observed in control bees may be explained by influences of the allatectomy operation that are not mediated by JH, or by relatively low sensitivity of the wax glands to JH (and therefore a higher JH dose is needed for a full recovery). JH appears to be involved in regulating an additional exocrine function, the chemistry of the Dufour's gland. The Dufour's gland has been implicated in various functions in honey bees including caste specific pheromones [54], [55] and fertility signaling [56]–[58]. The levels of esters in the secretion is positively associated with ovary development in reproductive workers [56]. In contrast in *B. terrestris* high levels of esters in the Dufour's glands are positively correlated with low reproductive state [40], [59], [60]. Our findings that the Dufour's glands of allatectomized bees contained a higher proportion of esters compared to the control and the sham operated bees (Fig. 6) suggest that JH influences the ester-biosynthetic activity of the Dufour's glands. Taken together our experiments support the idea that JH coordinates the function of diverse tissues and multiple physiological systems related to reproduction in bumblebees. This is similar to JH functions in other insects in which it is a principal gonadotropin [7], [10].

The powerful manipulation of circulating JH levels by allatectomy and replacement therapies allows us, for the first time, to comprehensively compare JH functions in adult honey bees (*A. mellifera*) and bumblebees (*B. terrestris*). The most obvious difference between the two bee species is the influence of JH on female fertility. Whereas we clearly show here that JH is necessary for oocyte development and reproduction in the bumblebee, similar manipulations of JH levels did not affect the fertility of adults female honey bees (see Introduction; [12], [14], [15]. These findings also contrast with studies showing that in adults honey bees JH represses *Vg* levels [17]–[19]. In the honey bee there is evidence suggesting that the main physiological function of *Vg* in workers is the regulation of nursing behavior rather than fertility. These include *Vg* functioning as an endocrine signal and as a rich protein source for royal jelly production in the hypopharyngeal glands [18]. An additional difference is in the influence of JH on wax secretion and processing. By contrast to our findings suggesting that JH influences wax secretion in *B. terrestris*, in the honey bee similar allatectomy and JH-III treatments did not affect the onset of wax production or the amount of wax produced [61]. The two species also appear to differ in the involvement of JH in the regulation of division of labor. In honey bees high JH levels are correlated with foraging behavior; treatments with JH, JH analogs, or JH mimics accelerates, whereas allatectomy delays the transition from nursing to foraging activities, [21], [22], [41], [62]. The influence of JH on division of labor in bumblebees has not been studied with similar details, but the available literature [35], [36], and our unpublished results (Siegel, Shpigler, Huang, and Bloch, unpublished data) suggest that JH does not influence foraging or nursing activities in *B. terrestris*.

Not all JH influences differ between the bumblebee and the honey bee. For example, in both species JH augments the expression of the transcription factor *Kr-h1*. Our study showing that JH upregulates *Kr-h1* expression in the bumblebee fat body is consistent with previous studies showing similar upregulation in the brain of both the honey bee and *B. terrestris* [33], [63], [64]. It is also interesting to note that despite the many differences in the influence of JH on the social physiology of *B. terrestris* and *A. mellifera*, the environmental regulation of JH titers show notable similarities [2]. The presence of the queen suppresses JH biosynthesis and hemolymph titers in workers of both the honey bee [65], [66] and the bumblebee [28], [43], [44], [48], [67]. In both species JH levels in young workers are also inhibited in the presence of older (*A. mellifera*; e.g., [68]), or dominant (*B. terrestris*; [29], [49], [69]) workers.

The evolution of complex traits such as those associated with advanced eusociality may require numerous modifications in multiple tissues, and in pathways controlling morphological, physiological, and behavioral processes. The integrative and coordinative nature of the endocrine system makes it very suitable for accommodating these profound changes that may need to occur over a relatively short evolutionary period. Our study showing that in contrast to the honey bee, in the bumblebee *B. terrestris* JH regulates reproductive physiology, lends credence to earlier suggestions that evolution of advanced eusociality in honey bees was associated with major modification in JH signaling [5], [24]–[26]. Our study sets the stage for unveiling the molecular underpinnings of these evolutionary modifications in JH signaling pathways. The modification in JH signaling along the evolution of advanced sociality may not be unique to honey bees because there is evidence for an association between JH levels and division of labor rather than ovarian state also in advance eusocial ants and wasps in which eusociality evolved independently from bees [2].

## Supporting Information

Figure S1 **The survival of bees in Experiment 1–3.** Day 1 is the day of dissection; the bees that survived the first day were divided into groups on day 2. On day 7 the bees were collected for analysis. CA-  =  allatectomized bees; Sham  =  sham operated bees; CA-+JH  =  CA- bees with replacement therapy. The plot shows that most of the mortality of allatectomized bees occurred on the first day after dissection.
(TIF)

Figure S2 **The influence of allatectomy on oogenesis in Experiment 1.** The values are mean ± SE, sample size within or above bars. For additional details see Fig. 2.
(TIF)

Figure S3 **The influence of allatectomy on wax secretion in Experiment 1.** **A**. The amount of wax secreted; **B**. the number of wax pots and cells constructed in a cage during the experiment. Graph details as in Fig. 5.
(TIF)

Table S1 **The body size of bees in experiments 1–3.**
(DOCX)

Table S2 **Sequences of primers used for qPCR.**
(DOCX)
