# Supplementary figures and images for: Gonadotropic and Physiological Functions of Juvenile Hormone in Bumblebee (Bombus terrestris) Workers
Source: PLoS One. 2014 Jun 24;9(6):e100650. doi: 10.1371/journal.pone.0100650 (PMC4069101; doi:10.1371/journal.pone.0100650)

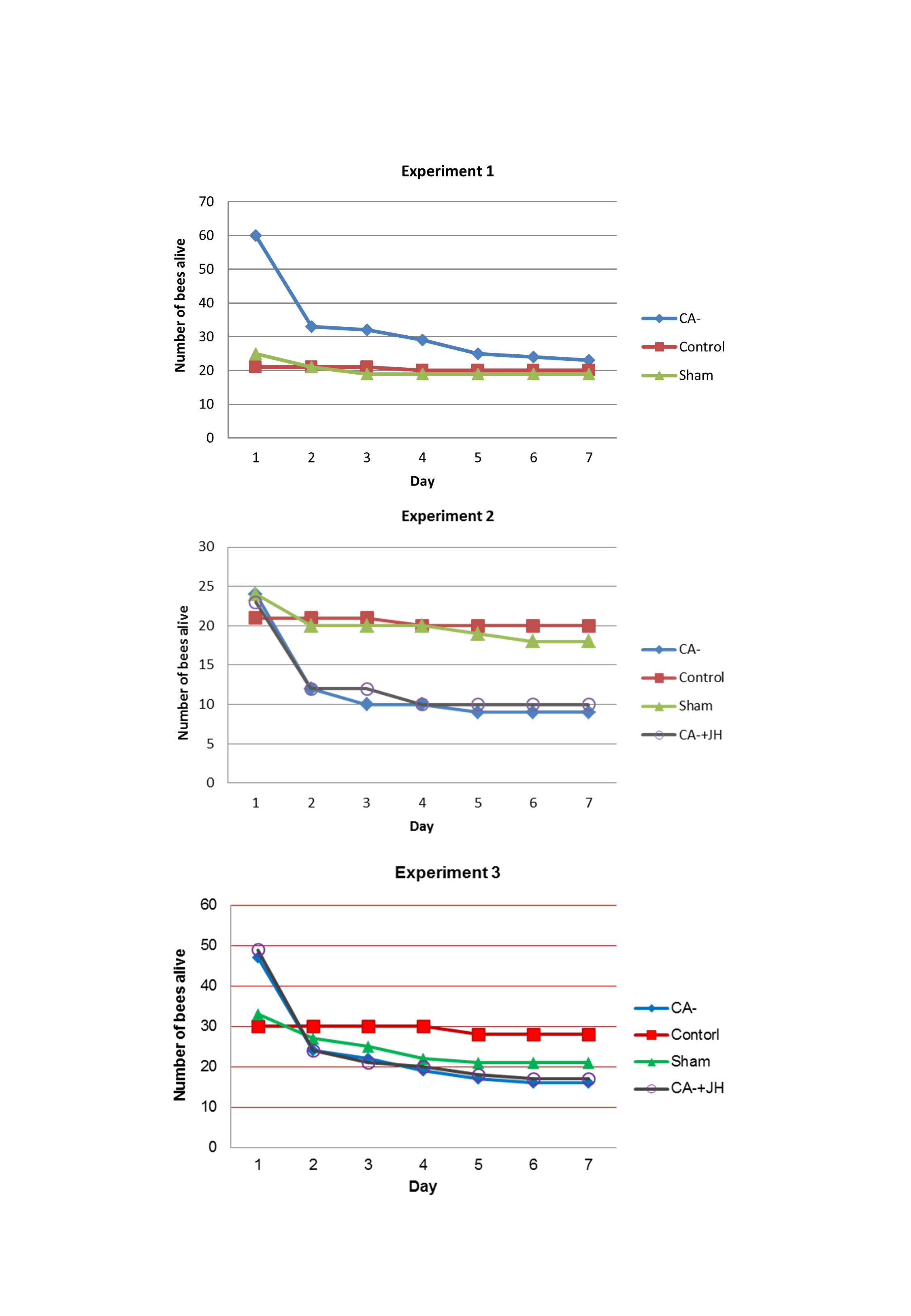

Supplement: Figure S1 — The survival of bees in Experiment 1–3. Day 1 is the day of dissection; the bees that survived the first day were divided into groups on day 2. On day 7 the bees were collected for analysis. CA- = allatectomized bees; Sham = sham operated bees; CA-+JH = CA- bees with replacement therapy. The plot shows that most of the mortality of allatectomized bees occurred on the first day after dissection. (TIF) [file pone.0100650.s001.tif]
