# Supplementary material for: Gonadotropic and Physiological Functions of Juvenile Hormone in Bumblebee (Bombus terrestris) Workers
Source: PLoS One. 2014 Jun 24;9(6):e100650. doi: 10.1371/journal.pone.0100650 (PMC4069101; doi:10.1371/journal.pone.0100650)
Supplement: Table S1 — The body size of bees in experiments 1–3. (DOCX) [file pone.0100650.s004.docx]

Supp. Table 1: The body size of bees in experiments 1-3.

|  | **CA-** | **Control** | **Sham** | **CA- + JHIII** | **p-value** |
| --- | --- | --- | --- | --- | --- |
| Exp. 1 | 2.96±0.08mm (10) | 2.89±0.08mm (7) | 3.0±0.05mm (15) |  | **0.51** |
| Exp. 2 | 3.05±0.08mm (7) | 3.08±0.04mm (15) | 3.05±0.03mm (15) | 2.96±0.07mm (5) | **0.35** |
| Exp. 3 | 2.94±0.04mm (15) | 2.94±0.05mm (27) | 2.97±0.05mm (26) | 2.88±0.04mm (16) | **0.64** |

Data represent mean ± SE marginal cell length for each experimental group. Sample size is in brackets. p-value was obtained from one-way ANOVA.
