# Supplementary material for: Gonadotropic and Physiological Functions of Juvenile Hormone in Bumblebee (Bombus terrestris) Workers
Source: PLoS One. 2014 Jun 24;9(6):e100650. doi: 10.1371/journal.pone.0100650 (PMC4069101; doi:10.1371/journal.pone.0100650)
Supplement: Table S2 — Sequences of primers used for qPCR. (DOCX) [file pone.0100650.s005.docx]

Supp. Table 2: Sequences of primers used for qPCR

| Primer name | Primer sequence |
| --- | --- |
| *BtVg*-F | CGGAATTTGTCGCCCGCCTTC |
| *BtVg*-R | GCAGGAAAGGATGGACGACGAGG |
| *BtKrh-1*-F | CGCCGGATCCTCTCGAT |
| *BtKrh-1*-R | TTTTCTTGGAGGGAGAATTAACGT |
| *BtEf1a*-F | CGTTTACCGCTTCAGGACGT |
| *BtEf1a*-R | GCATGCCTGGTTTCAGAATACC |
